# Biogas Production from Sunflower Head and Stalk Residues: Effect of Alkaline Pretreatment

**DOI:** 10.3390/molecules25010164

**Published:** 2019-12-31

**Authors:** Marinela Zhurka, Apostolos Spyridonidis, Ioanna A. Vasiliadou, Katerina Stamatelatou

**Affiliations:** Department of Environmental Engineering, Democritus University of Thrace, 67100 Xanthi, Greece; maria123zhurka@hotmail.com (M.Z.); aspyri@env.duth.gr (A.S.); ioavasil@env.duth.gr (I.A.V.)

**Keywords:** anaerobic digestion, lignocellulosic biomass, NaOH pretreatment, bioreactor experiments, biochemical methane potential, inhibition

## Abstract

Sunflower residues are considered a prominent renewable source for biogas production during anaerobic digestion (AD). However; the recalcitrant structure of this lignocellulosic substrate requires a pretreatment step for efficient biomass transformation and increased bioenergy output. The aim of the present study was to assess the effect of alkaline pretreatment of various parts of the sunflower residues (e.g., heads and stalks) on their methane yield. Experimental data showed that pretreatment at mild conditions (55 °C; 24 h; 4 g NaOH 100 g^−1^ total solids) caused an increase in the biochemical methane potential (BMP) of both heads and stalks of the sunflower residues as determined in batch tests. The highest methane production (268.35 ± 0.11 mL CH_4_ g^−1^ volatile solids) was achieved from the pretreated sunflower head residues. Thereafter; the effect of alkaline pretreatment of sunflower head residues was assessed in continuous mode; using continuous stirred-tank reactors (CSTRs) under two operational phases. During the first phase; the CSTRs were fed with the liquid fraction produced from the pretreatment of sunflower heads. During the second phase; the CSTRs were fed with the whole slurry resulting from the pretreatment of sunflower heads (i.e., both liquid and solid fractions). In both operating phases; it was observed that the alkaline pretreatment of the sunflower head residues had a negligible (phase I) or even a negative effect on biogas production; which was contradictory to the results of the BMP tests. It seems that; during alkaline pretreatment; this part of the sunflower residues (heads) may release inhibitory compounds; which induce a negative effect on biogas production in the long term (e.g., during continuously run digesters such as CSTR) but not in the short-term (e.g., batch tests) where the effect of the inoculum may not permit the inhibition to be established.

## 1. Introduction

Lignocellulosic substrates are considered important feedstocks for the production of second-generation biofuels (e.g., H_2_, CH_4_). Agricultural residues comprise a renewable resource which does not compete with plant cultivation for food, since they consist of the nonedible parts of the plants (leaves, stalks), which are usually burned on the fields creating environmental issues related with biomass incineration in the open air [1]. This is also the case with sunflower residues (stalks, leaves, heads) which are produced in large quantities and left in the fields after seed harvesting. In the absence of any alternative reuse, sunflower residues may be a promising renewable resource for bioenergy production via anaerobic digestion [2]. However, the presence of lignin is apparently the most important factor affecting the biodegradability of lignocellulosic materials. Therefore, the recalcitrant structure of lignocellulosic substrates consisting of holocelluloses (cellulose, hemicelluloses) that are embedded in the lignin network, requires the application of pretreatment, to increase the accessibility of holocelluloses to bacteria during anaerobic digestion [3].

Pretreatment methods, such as mechanical, thermo-chemical, chemical, and biological, have been applied to improve biogas production from anaerobic digestion of lignocellulosic residues [4,5]. Among them, dilute-acid and alkaline chemical pretreatments are the most widely used for sunflower residues, being low-cost and effective processes [5,6,7]. Dilute-acid pretreatment, hydrolyzes hemicelluloses into monomeric sugars, improving cellulose conversion and increasing enzyme accessibility [8]. By alkaline pretreatment, the cleavage of ester bonds mainly occurs in lignin/phenolics–carbohydrate complexes, resulting in partial lignin removal [3]. On the other hand, it should be emphasized, that applying pretreatment on plant biomass, lignin derived compounds are released which may be inhibitory to anaerobic microorganisms [6]. Acid pretreatment causes the hydrolyzation of the hemicellulosic fraction into furaldehydes [5-hydroxymethylfurfural (5-HMF) and furfural] and aliphatic acids (i.e., formic and acetic acid) [9]. Moreover, Antonopoulou et al. [7] reported that high NaOH concentration during pretreatment of sunflower straw biomass, resulted in a high concentration of phenolics. These compounds might have an inhibitory or even toxic effect on anaerobic microorganisms and therefore, should be always taken into consideration. Monlau et al. [6,10] applied various chemical pretreatments on sunflower straw studying their influence on lignin removal and evaluating the biochemical methane potential (BMP). They reported that alkaline pretreatment at 55 °C, for 24 h with 4 g NaOH 100 g^−1^ total solids, was the most suitable for enhancing the anaerobic digestion process and methane potential. Monlau et al. [2], applied this mild NaOH pretreatment in continuous experiments and noticed an increase of 26% in methane yield. However, it is not well understood how the different parts of the residues (i.e., stalks and heads) can respond to the pretreatment scheme at different modes of the anaerobic digestion process (batch and continuous).

The case study considered in this work was of the sunflower residues, abundant in Greece and especially in the area of East Macedonia and Thrace. FAOSTAT recorded an area of 90,600 hectares of sunflower seeds cultivated in Greece, with an estimated yield of 2433.8 kg/ha in 2017 [11]. These agricultural areas produce large quantities of sunflower residues; Searle and Malins [12] estimated that the ratio of residue to harvested grain is 1.77 for the case of sunflower crops. Therefore, they could be used as feedstock in biogas plants which have been developed in Greece over the recent decade. The biochemical methane potential of an organic material is usually evaluated through batch tests. On the other hand, experimental results coming from continuously run digesters are scarce but more trustworthy since they simulate the conditions prevailing in real biogas plants (which operate on a continuous mode) and reveal the long-term response of the anaerobic biomass to a particular feedstock. To this end, the effect of alkaline pretreatment on the biogas production from sunflower residues was studied in two different types of digesters (batch and continuous), focusing mostly on the heads. Consequently, the results of the presented study can be considered to be complementary to those reported in Monlau et al. [2], which is the only reference (to our knowledge) focusing on the digestion of sunflower stalks in continuous mode at mesophilic conditions, after alkaline pretreatment under the same conditions. These results show how an estimation based on the typical biomethane potential tests can be proven wrong by experiments run in continuous mode.

## 2. Results and Discussion

### 2.1. Sunflower Heads and Stalks Characteristics

Heads and stalks, prior to any analysis, were dried in an oven resulting in a low humidity level of ca. 10% for both residues. The chemical oxygen demand (COD) concentration was higher for stalks by 20.8% in comparison to heads, which is in agreement with the higher volatile solid (VS) content of stalks (87.7% of total solids; TS for heads and 79.9% of TS for stalks). On the contrary, total Kjeldahl nitrogen (TKN) and lipids of heads were more than two times higher compared to stalks (Table 1). 

The latter could be attributed to some seed residues in the heads, which are rich in lipids and proteins [13]. Klason lignin content of raw stalks reported here (20.36% VS, Table 2) was lower compared to the results of Monlau et al. (29.7% VS) [6]. Klason lignin concentration of stalks (20.36% VS) was much higher in comparison to heads (10.48% VS, Table 2), providing a more rigid structure for stalks and reducing their biodegradability. Elemental analysis showed that both, heads and stalks, contained a significant amount of Ca, Mg, K, Si, and Fe (Table 3). Nevertheless, trace elements such as Co, Mo, Ni, and W which are considered to be essential for the process of anaerobic digestion [14], were either detected in small concentrations or were absent.

### 2.2. Pretreatment Effect on the Biochemical Methane Potential (BMP) of Sunflower Heads and Stalks—Batch Experiments

**Figure 1:**
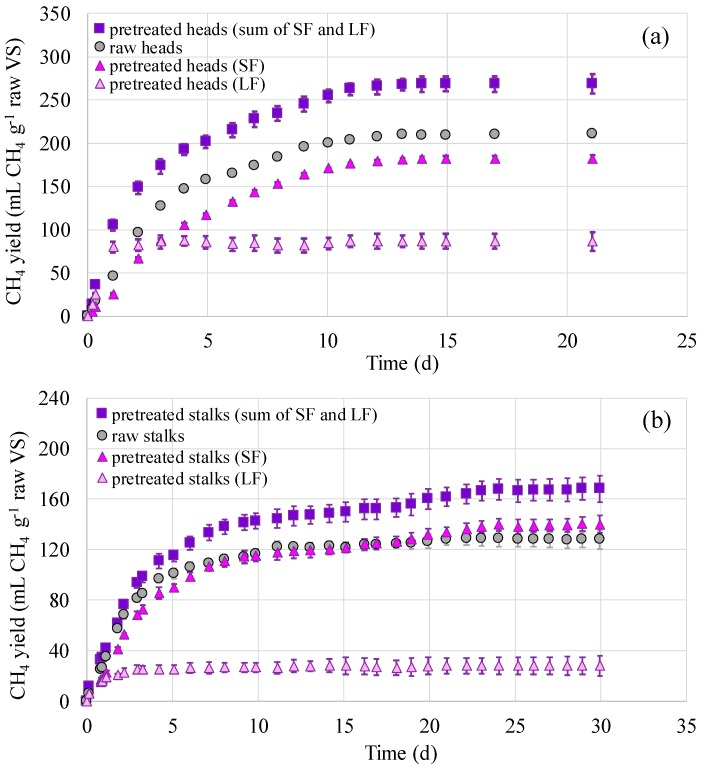
Biochemical methane potential (BMP) profiles of pretreated and untreated sunflower (**a**) heads and (**b**) stalks. Values correspond to means of triplicates of independent values ± standard deviations (error bars). SF: solid fraction, LF: liquid fraction.

The BMPs of all fractions were expressed as mL CH_4_ per g of vs. prior to pretreatment (called as “raw”) for both pretreated and untreated sunflower heads and stalks, so that comparisons were possible. Control tests were conducted with the untreated sunflower head or stalk (raw). In all cases, the methane volume produced from the blank tests was subtracted to exclude the contribution of the inoculum in the BMP. The alkaline pretreated sunflower head or stalk were separated into their respective liquid and solid fractions (LF and SF respectively). Each fraction was tested separately, but since the BMP of each fraction was expressed per vs. of the raw material it came from, the BMPs of both LF and SF could be summed up and, therefore, express the total BPM of the pretreated head or stalk. The results are shown in Figure 1. In all cases, the LF yielded the lowest methane but at the highest rate. This was attributed to the less, but more readily available organic matter present in the LF as compared to the SF.

Focusing on the untreated residues (raw heads and stalks), it can be seen that the BMP of raw stalk was 65% lower (*p* = 1.4 × 10^−5^, <0.05) compared to the raw head residues, i.e., 127.98 ± 5.19 and 210.56 ± 1.97 mL CH_4_ g^−1^ raw VS, respectively (Table 4). This was not expected since the heads contained less VS, resulting in less COD (Table 1). However, their different lipid content (2.5 g kg^−1^ TS heads vs. 0.99 g kg^−1^ TS stalks) and protein content (13.3 g TKN kg^−1^ TS heads vs. 6.3 g TKN kg^−1^ TS stalks) could explain the higher BMP of the heads (lipids and proteins yield higher methane than other organic compounds such as carbohydrates). Similarly, after alkaline pretreatment, the methane yield of sunflower stalks was estimated to be 168.17 ± 6.87 mL CH_4_ g^−1^ raw VS, which was significantly lower (*p* = 2.2 × 10^−5^, <0.05) than the BMP of pretreated sunflower heads (268.47 ± 3.38 mL CH_4_ g^−1^ raw VS) (Table 4). In fact, it was 37% lower. The methane yield achieved from the pretreated heads was 60% higher than the yield from the pretreated stalks (Table 4).

In general, alkaline pretreatment as resulted from the BMP tests enhanced the methane yield by 31% and 28% for the stalk and head residues, respectively. This can be correlated with the lignin removal (Table 2) observed after alkaline pretreatment of head and stalk residues (31% and 8%, respectively). Similar results were obtained by Monlau et al. [15] who applied alkaline pretreatment under the same conditions and observed a high lignin removal of 22%, after pretreatment at 55 °C. Moreover, the lignin removal achieved in this study for sunflower heads is comparable with the removal achieved under higher temperature conditions in other studies. Antonopoulou et al. [7] reported 20% and 36% of lignin removal using 2 and 20 g NaOH 100 g^−1^ TS, respectively, at 80 °C.

Finally, it is noteworthy that Monlau et al. [6] achieved higher methane potential from sunflower stalks (259 ± 6 mL CH_4_ g^−1^ raw VS) as compared to this study (Table 4), after pretreatment at 55 °C with 4% NaOH for 24 h. In another study, Monlau et al. [10] found a methane potential of 262 mL CH_4_ g^−1^ raw vs. from sunflower stalks using identical alkaline pretreatment. The BMP of the untreated sunflower stalks was also higher than this study (193 mL CH_4_ g^−1^ raw vs. > 128 mL CH_4_ g^−1^ raw VS). Furthermore, Hesami et al. [16] reported much higher values, compared to this study, BMPs from sunflower stalks after hydrothermal (180 °C, 60 min) and isopropanol-based organosolv pretreatment (160 °C, 30 min, 1% H_2_SO_4_), of 234 and 278 mL CH_4_ g^−1^ VS, respectively. However, the methane production yield obtained from the digestion of untreated stalks was lower (124 mL CH_4_ g^−1^ VS) than this study. The differences in BMPs as reported in various research studies should be expected, due to the differences in the sunflower variety, the geographical area and the practices used to grow the crop [17]. This is in agreement with Monlau et al. [10], who reported that the alkaline pretreatment (55 °C, 24 h, 4 g NaOH 100 g^−1^ TS) of stalks of different sunflower varieties resulted in different lignin reduction, varying from 23.3% to 36.3% VS. Moreover, the time the residues were left in the field after harvesting and prior to collection is also a determining factor. In the case of the present study, the residues were collected approximately 2–3 weeks after harvesting.

### 2.3. Continuous Stirred Tank Reactors (CSTRs)–Continuous Experiments

BMP tests indicated that pretreated head residues were the most promising substrate for methane production. Therefore, continuous experiments were performed to evaluate the long-term effect of alkaline pretreatment on biogas production from sunflower head residues. During the first operational phase, all three CSTRs were fed on the liquid fraction coming from the pretreated sunflower head (CSTR1 and CSTR2) or the untreated sunflower head under the conditions summarized in Table 5. 

**Figure 2:**
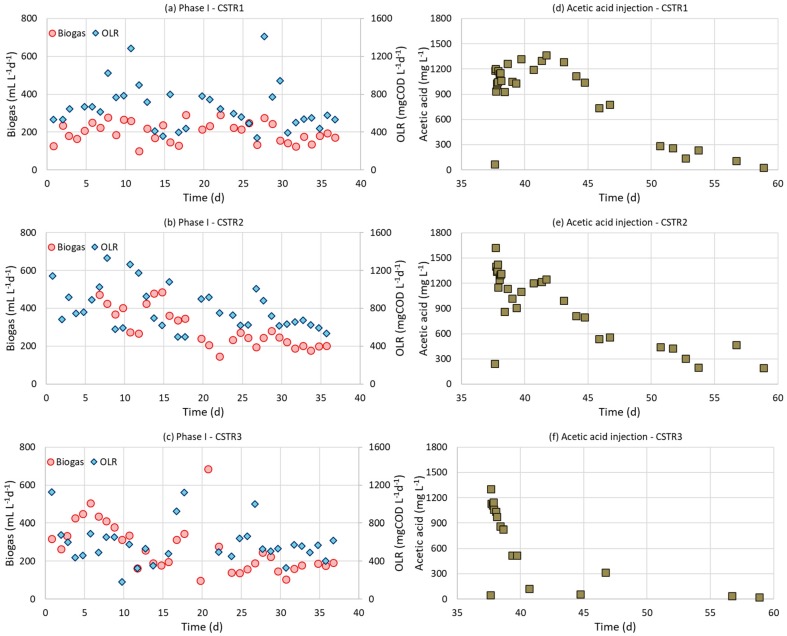
Biogas production rate compared to organic loading rate in three CSTRs fed on sunflower heads during operational phase I (**a**) CSTR1 (NaOH pretreated, HRT = 21 ± 7 d), (**b**) CSTR2 (NaOH pretreated, HRT = 15 ± 4 d), (**c**) CSTR3 (untreated, HRT = 16 ± 6 d) and acetic acid degradation profiles after adding acetate on the 37th day in (**d**) CSTR1, (**e**) CSTR2, (**f**) CSTR3.

The organic loading rates (OLRs) and biogas production rates (BPRs) versus time are shown in Figure 2a–c. The experimental data showed a significantly higher biogas production rate during the operation of CSTR2 (205 ± 23 mL L^−1^ d^−1^) than CSTR1 (161 ± 26 mL L^−1^ d^−1^) (*p* = 0.005, <0.05) (Table 6), due to the higher OLR in CSTR2. However, the methane yield achieved by CSTR2 (0.193 ± 0.035 L CH_4_ g^−1^ VS) was comparable with the yield achieved by CSTR1 (0.187 ± 0.025 L CH_4_ g^−1^ VS) (*p* = 0.71, >0.05). The concentration of phenolic compounds determined in both CSTRs during operation was below 250 mg L^−1^ (data not shown).

On the other hand, despite the alkaline pretreatment of the feeding substrate in CSTR2, the biogas production rate of CSTR2 (205 ± 23 mL L^−1^ d^−1^) was not significantly higher than the control CSTR3 (179 ± 12 mL L^−1^ d^−1^) (Table 6) that operated at the same HRT (16 ± 6 d) (*p* = 0.67, >0.05). In order to elucidate if any inhibition occurred in the bioreactors, 1 g of acetic acid per L of reactor was added in all three CSTRs, while the feeding with the liquid fraction of the sunflower heads continued. This disturbance occurred on the 37th day when the biogas had been stabilized in all three CSTRs. Acetic acid remained constant with some temporal increases in the CSTR1 and CSTR2 (Figure 2d,e). This indicated that no more acetic acid than that produced from the digestion of the liquid fraction could be degraded, which revealed that these reactors were kinetically limited. On the other hand, the acetic acid was degraded in the CSTR3 within 5.5 days (Figure 2f). To alleviate the inhibitory effect that averted acetic acid degradation, the feeding of CSTR1 and CSTR2 was stopped and these reactors operated at batch mode until acetic acid concentration was minimized. Even at batch mode, the degradation of acetic acid was much slower in CSTR1 (84 mg L^−1^ d^−1^) and CSTR2 (50 mg L^−1^ d^−1^) than in CSTR3 (350 mg L^−1^ d^−1^).

**Figure 3:**
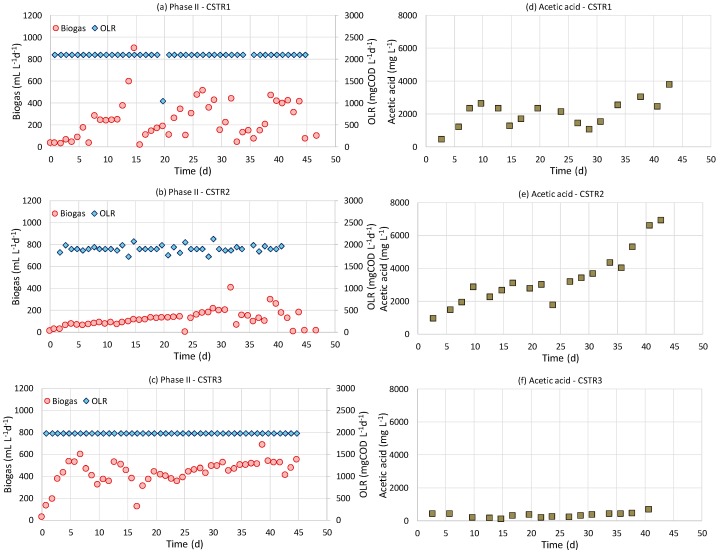
Biogas production rate compared to organic loading rate in three CSTRs fed on sunflower heads during operational phase II (**a**) CSTR1 (NaOH pretreated, 4% NaOH), (**b**) CSTR2 (NaOH pretreated, 8% NaOH), (**c**) CSTR3 (untreated) and acetic acid degradation profiles in (**d**) CSTR1, (**e**) CSTR2, (**f**) CSTR3.

The CSTRs’ operation was continued with feeding of the pretreated sunflower head as a whole (without separating it into liquid and solid fractions). This enabled a direct comparison with the results of Monlau et al. [2], who also fed the CSTRs with the pretreated sunflower stalks as a whole. Moreover, it could be possible that feeding with a medium rich in solids would allow the slow release of organic matter due to hydrolysis, which may be beneficial. The solid matrix of the feeding material could also provide a more suitable microenvironment for the microorganism to cope with adverse conditions. Blika et al. [18] reported that the presence of solids in olive-mill wastewater was a crucial factor for the stability of the AD process, that might have resulted in adsorption of toxic hydrophobic compounds onto the solids, such as long-chain fatty acids. Therefore, during phase II, the three CSTRs were fed with an increased OLR of ac. 2000 mg L^−1^ d^−1^ (Table 5). All CSTRs were operated under an HRT of 25 days, while CSTR2 was fed on sunflower heads pretreated at a higher dose of NaOH (8 g 100 g^−1^ TS compared to 4 g 100 g^−1^ TS). Both CSTR 1 and CSTR2 were unstable, while CSTR3 exhibited a stable response in terms of biogas production rate (Figure 3). The biogas and methane yield were 0.26 ± 0.03 L g^−1^ vs. and 0.15 ± 0.015 L CH_4_ g^−1^ vs. in CSTR3, respectively. Monlau et al. [2] found that the methane yield of sunflower stalks without alkaline preferment was 0.152 L g^−1^ vs. (which agrees with the methane yield of the non-treated heads obtained in this study), while after pretreatment it was increased by 25.6% to 191 mL g^−1^ vs. (which does not agree with the results of the present study, since alkaline pretreatment caused instability in the digesters). The inhibitory conditions prevailed in the CSTR1 and the CSTR2 are also proven by the increasing trend of acetic acid profiles in these digesters which is more intense in the case of CSTR2 (Figure 3d,e). This indicates that inhibition is related with the NaOH pretreatment which was harsher in the feeding of CSTR2 (a double dose of NaOH, i.e., 8 g 100 g^−1^ TS, was applied). Eventually, CSTR3, which was fed with raw sunflower head residues, achieved the highest biogas production rate (505 ± 52 mL L^−1^ d^−1^) as compared to CSTR1 and CSTR2.

In another work, Polat et al. [19] studied the methane production of a lab-scale fermenters fed with a slurry (2% TS *w*/*v*) of alkali-pretreated sunflower heads (2 and 5 g NaOH 100 g^−1^ TS, 24 h, ambient temperature) at various HRTs (8−15 d) at thermophilic conditions. They found a positive effect of the alkaline pretreatment. The biogas and methane yields of sunflower heads without alkaline pretreatment were 0.199 L g^−1^ vs. and 0.125 L CH_4_ g^−1^ VS, respectively, for an HRT of 15 days. These values are lower than the yields found in this study for the control reactor (0.26 ± 0.03 L biogas g^−1^ vs. and 0.15 ± 0.015 L CH_4_ g^−1^ vs. in CSTR3), but it should be taken into account that the HRT was longer i.e., 25 d). However, when compared to the yields they obtained at a dose of 5 NaOH 100 g^−1^ TS, the yields were 20% higher (0.249 L biogas g^−1^ vs. and 0.154 L CH_4_ g^−1^ VS). It should be noted, that the solid concentration of the feeding slurry in Polat et al. [19] was lower (2%) than in this work (4.6%). Although the NaOH dose was a little higher (5 NaOH 100 g^−1^ TS) than the dose used in CSTR2 (4 NaOH 100 g^−1^ TS), the feeding in Polat et al. [19] contained less TS which may have induced a lower concentration of inhibitors. Moreover, the pretreatment in Polat et al. [19] was conducted at even milder conditions with respect to temperature. This might be an additional reason for less inhibitors’ production during alkaline pretreatment.

Regarding the causes of process low efficiency or instability while the digesters were fed on alkaline pretreated sunflower heads, it is usually considered that the solubilization or degradation of lignin results in secondary by-products such as phenolic or other compounds which may cause inhibition (i.e., vanillin, syringaldehyde) [2]. There are contradictory reports in the literature about their inhibitory level. For example, furfural and 5-hydroxymethylfurfural (HMF) which originate from the dehydration of pentoses and hexoses, as well as syringaldehyde and vanillin resulting from the lignin polymers’ degradation, were not found inhibitory (even at high concentrations, 1 g L^−1^), and, moreover, were converted to biogas during BMP tests [20]. On the other hand, Kayembe et al. [21] evaluated the inhibitory effect of phenolic monomers on methane production by acetoclastic methanogens (archaea) in batch assays, correlating the inhibition with the hydroxyl groups’ number of their aromatic structures. They suggested that monomers as phenol and resorcinol, exhibit inhibition of ca. 1.2 and 1.7 g L^−1^ IC50, respectively, corresponding to 50% inhibition of methanogenic activity. However, the phenolics measured in the liquid fractions of this study (Table 1) were low enough compared to the above IC50 values. This indicates that phenolics as measured through the Folin–Ciocalteu method are not the cause of inhibition, but this does not exclude the presence of other potential inhibitors resulting as derivatives from the sunflower heads after alkaline pretreatment.

Apart from the inhibitory compounds probably released during pretreatment, the higher sodium ion concentration, might also be a cause for methanogenic bacteria inhibition. Antonopoulou et al. [22] observed a threshold of NaOH (1% *w*/*v* NaOH, 0.5% TS *w*/*v*) concentration above which, inhibition or toxicity of methanogens might occur. This corresponds to a sodium cation concentration of 5750 mg L^−1^. Moreover, Polat et al. [19] reported an unstable behavior of continuously run digesters fed on sunflower heads when the solid concentration of the feed was 5%. They correlated this instability with the excessive alkali dosage (NaHCO_3_) required to adjust the very low pH of the feeding medium. A concentration range of sodium ion between 3500 and 5500 mg Na^+^ L^−1^ has been considered to cause moderate inhibition [23]. In the presented study, the sodium ion concentration entering the digesters was estimated to be 1058 and 2116 mg Na^+^ L^−1^ in the case of pretreatment with 4 g and 8 g NaOH per 100 g TS, respectively (4.6% TS was the influent concentration). Therefore, alkali pretreatment, at these NaOH doses applied in this work, does not seem to have caused inhibition through the sodium anion.

Finally, the contradictory results for the methane yields derived from batch and continuous experiments in the present study reveal that batch tests may not give representative results since they are affected by the activity of the inoculum. A highly active inoculum may achieve yield high ratios of methane produced per mass of substrate. On the contrary, under continuous operation, the inoculum is constantly exposed to the feeding conditions which increases the concentration of inhibitory compounds inside the reactor if these are recalcitrant and are not consumed. Therefore, alkaline pretreatment seems to have a positive effect due to organic matter solubilization and destruction of the lignocellulosic matrix, but also a negative effect due to inhibitory compounds probably released as lignin degrades [2]. The positive effect was prominent in the short term during the BMP tests, while the negative effect prevailed in the long term during continuous operation of the digesters.

## 3. Materials and Methods

### 3.1. Sunflower Residues Collection and Pretreatment

**Figure 4:**
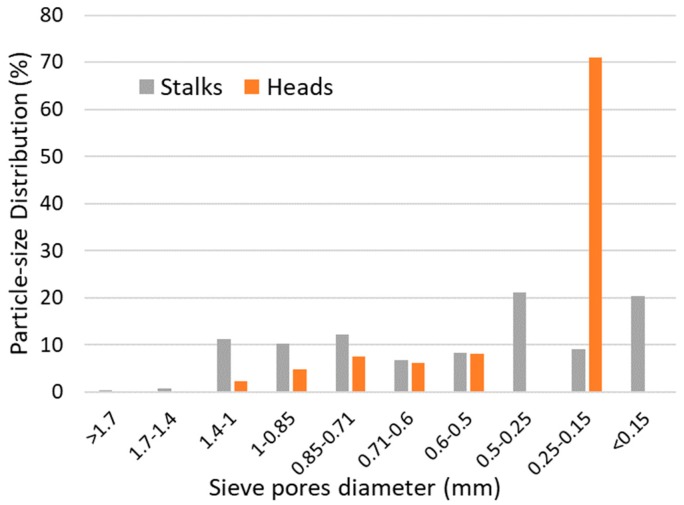
Graphical presentation of particle-size distribution of milled stalks and heads after sieving.

Sunflower residues were collected, from a farm located in northern Greece (Xanthi city) after seed harvest. The residues were separated into stalks and heads after seed removal. The heads and stalks were dried at 40 °C in an oven (MMM-Venticell) and then milled separately to obtain a particle size of ca. 0.15–1.4 mm using a cutting mill (Retsch SM100). The particle size distribution is shown in Figure 4. The alkaline pretreatment conditions (55 °C, 24 h, 4 g NaOH 100 g^−1^ TS) were selected after Monlau et al. [6]. A loading of heads or stalks equal to 4.6 g dry matter was added into glass bottles (120 mL) with a working volume of 100 mL. The bottles were continuously stirred in a thermostatically controlled incubator at 55 °C. After pretreatment, the pH value was adjusted to 7 with 6N HCl and the whole slurry was separated into liquid and solid fractions by filtration through a 2.5 mm mesh filter. The solid fractions were dried at 40 °C for 24 h before analysis. The main characteristics of the raw and pretreated fractions of the sunflower residues are summarized in Table 1 as average values from triplicate analysis. Table 2 shows the lignin content (Klason lignin) of raw and pretreated heads and stalks. Klason lignin or acid-insoluble lignin is the insoluble residue portion obtained after removing the ash by concentrated acid hydrolysis of the plant tissues [24].

### 3.2. Biochemical Methane Potential

In order to evaluate the biochemical methane potential (BMP) of the pretreated residues, the solid and liquid fractions of the heads and the stalks were separately digested in batch anaerobic serum bottles (110 mL, working volume 97.5 mL). All BMP tests were conducted in triplicate at 37 °C. Each bottle was inoculated with 85 mL of anaerobic sludge (24.3 g vs. L^−1^) as inoculum, taken from a full-scale anaerobic digester fed on cow manure and a mixture of various wastes. The inoculum was kept for about 5 days before the batch digestion test at 37 °C for degassing. Then 12.5 mL of the liquid fraction or 1.07 g of the solid fraction were added into serum bottles to achieve an organic loading of 0.04 or 0.55 g COD g^−1^ vs. inoculum, respectively. Since the liquid fractions contained readily biodegradable COD, the COD loading was lower in these tests. On the other hand, the solid fractions contained organic matter, a part of which was biodegradable, and therefore, the COD added was higher. Control tests were run with raw (untreated) sunflower heads and stalks (organic loading of 0.55 g COD g^−1^ vs. inoculum). Blank tests containing only the inoculum and water to reach the same final volume were also set up. No addition of macro elements or trace elements took place. After the preparation of serum bottles, degasification with N_2_/CO_2_ mixture (80/20) was carried out for 1 min to obtain anaerobic conditions and the bottles were sealed with rubber stoppers. The headspace of each bottle was connected with a NaOH (6N) displacement apparatus to trap CO_2_. Methane production was monitored via the volume displacement method and was expressed at standard conditions of temperature and pressure (STP).

### 3.3. Continuously Stirred Tank Reactors: Set-up and Operation

**Figure 5:**
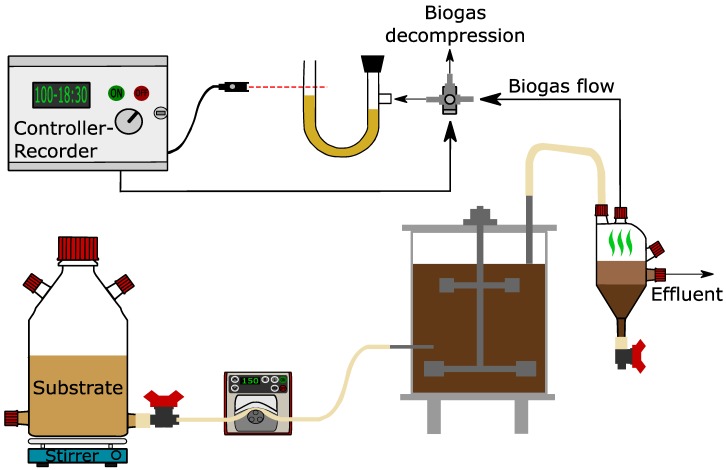
Experimental configuration used for the continuous tests.

Experiments were conducted in three identical continuously stirred tank reactors (CSTRs) (Figure 5). Each CSTR had 3.2 L and 2.5 L total and working volume, respectively. All reactors were constructed from two Plexiglas cylinders concentrically configured to allow for the recirculation of hot water within the in-between void space, thus maintaining the digester’s temperature at 35 ± 1 °C through a controller. The CSTRs were mixed at ca. 85 rpm by a mechanical stirrer attached onto the top of the reactors. The biogas and the effluent were removed from the same tube (which determined the level of the mixed liquor in the CSTR) into a flask and they were separated. This flask allowed the solids to settle down while the supernatant overflowed through a port placed in the middle. The solids were removed daily from the bottom of the flask. Moreover, the biogas was released from the upper part of the flask and was led to a volumetric measurement device consisting of a U-tube filled with oil and a 3-way valve solenoid. The valve was activated by a level sensor which allowed for the displacement of oil by the biogas entering the U-tube and the release of biogas after the oil reached a certain level (Figure 5). The biogas production rate (BPR) is reported at STP [25].

The three CSTRs were inoculated with biomass taken from a full-scale anaerobic digester treating various animal wastes. The operation can be distinguished in two main phases. As shown in Table 5, during the first operating phase, all three CSTRs were fed on the liquid fraction obtained from the sunflower residues’ heads as described in Section 2.1 in the presence of NaOH (mild alkaline pretreatment; CSTR 1 and CSTR 2) or in the absence of NaOH (control experiment; CSTR3). For the hydraulic retention time (HRT) it was attempted to maintain equality for CSTR2 and CSTR3, while the HRT was kept higher in the CSTR1 to study the effect of the feeding rate. Since feeding was conducted three times a day via peristaltic pumps set by timers, there was an inevitable variation in the feeding rate which was recorded daily. As a result of the different HRT and the liquid fraction’s characteristics (in the presence or absence of NaOH), the organic loading rate (OLR) was higher in the CSTR 2 (lower HRT) and lower in the CSTR3 (the absence of NaOH resulted in lower extraction of the organic matter in the liquid fraction) The liquid fraction was supplemented with 10 ml L^−1^ from nutrient and trace elements solutions consisting of 8.2 g L^−1^ K_2_HPO_4_ (solution A), 40.9 g L^−1^ NH_4_Cl (solution B), 33 g L^−1^ FeCl_3_.4H_2_O (solution C), and other trace elements (solution D; [25]) which were stored in a different bottle from Fe salt to avoid insoluble salt formation.

After the three CSTRs reached a quasi-steady biogas production rate under the operating conditions of phase I, an impulse disturbance of acetate was imposed, while the CSTRs continued to be fed with the sunflower substrate. Specifically, 1 g L^−1^ acetate was spiked simultaneously into all three CSTRs on the 37th day, increasing instantly the concentration of acetate inside the reactors. The aim of this disturbance was to study the response of the acetoclastic methanogens prevailing under the imposed operating conditions.

After depletion of acetate, the second operating phase started to introduce the whole slurry biomass (without separating it into liquid and solid fractions) after alkaline pretreatment in two different NaOH doses (CSTR1 and CSTR2) or without alkaline pretreatment (CSTR3; control experiment). Similarly, to the first operating phase, nutrient and trace element solutions were added. The feeding was conducted manually to avoid clogging problems due to the solids. The HRT resulting was 25 d for all three CSTRs. The organic loading rate (OLR) resulted as shown in Table 5. All feeding mixtures introduced into the CSTRs were adjusted to pH 7 with 6 N HCl.

### 3.4. Statistics

All data obtained were statistically analyzed to calculate average values (Equation (1)) and standard deviations (Equation (2)).

(1) $$\bar{Y}=\frac{\sum_{i=1}^{n}Y_{i}}{n}$$

(2) $$\mathrm{SD}=\sqrt{\frac{\sum_{i=1}^{n}{\left(Y_{i}-\bar{Y}\right)}^{2}}{n-1}}$$

where $\bar{Y}$ is the average of the measured values, SD is the standard deviation, $Y_{i}$ are the experimental values, and n is the number of data.

The 95% Confidence Interval (CI) on the mean was computed as follows (Equation (3)):

(3) $$\mathrm{CI}=\bar{Y}\pm Z_{95}\cdot \frac{\mathrm{SD}}{\sqrt{n}}$$

where Z_95_ is the upper (1 − 0.95)/2 critical value for the standard normal distribution.

An F-test was performed to determine the homogeneity of variances, followed by analysis of variance method (two-sample *t*-test, *p* 0.05), to assess the effect of pretreatment of sunflower residues on anaerobic digestion performance using Microsoft Excel software.

### 3.5. Analytical Methods

The characterization of sunflower heads and stalks as well as the samples from all bioreactors was performed in the laboratory of Wastewater Management and Treatment Technologies of the Department of Environmental Engineering at Democritus University of Thrace (DUTH, Greece). Chemical oxygen demand (COD), total Kjeldahl nitrogen (TKN), total solids (TS), volatile solids (VS) and lipids were determined according to Standard Methods [26]. The pH was measured with a pH meter (HAΝΝA, HΙ 83141, HANNA INSTRUMENTS HELLAS, Athens, Greece). Phenols’ concentration was measured according to Cindric et al. [27]. Klason lignin content was determined according to the Laboratory Analytical Procedure (LAP) [28]. The concentrations of volatile fatty acids (VFAs) were measured in a gas chromatograph (Perkin Elmer, Waltham, MA, US) equipped with a capillary free fatty acid phase (FFAP) column, a flame ionization detector, and helium as carrier gas, while the oven temperature program was 50 °C to 200 °C with 10 °C min^−1^ rate. A gas analyzer equipped with CO_2_ and CH_4_ infrared sensors (GasCard NG model of the Edinburgh Sensors) (Edinburgh Instruments Ltd, Livingston, UK) was used for determining biogas composition.

For the elemental analysis of stalks and heads, the samples were pelletized in the Atlas 25 ton Manual Hydraulic Press (Specac, Kent, UK) with Atlas™ 40 mm Evacuable Pellet Die (Specac, Kent, UK) by using a 2 g subsample with boric acid as substrate (H_3_BO_3_ 99.9%, Socachim, Belguim). Analysis was conducted using a Wavelength dispersive X-ray fluorescence spectrometer (ZSX Primus II, Rigaku Corporation, Tokyo, Japan), which was calibrated using the NIST standards: 1646a, 1648a, 2584, and 2710a.

## 4. Conclusions

Results presented herein showed that the sunflower head and stalk residues after alkaline pretreatment had a positive effect on the biogas production as evaluated during BMP tests. On the other hand, CSTR operation with pretreated sunflower heads, indicated the opposite. Experimental observations revealed that alkaline pretreatment obviously causes inhibitory conditions, which could not be detected in the batch tests where, mostly, the short-term response of the microorganisms is shown. This indicates, that BMP tests cannot always reveal the real effect of treatment conditions, since the microbial biomass is not continuously exposed to the positive or adverse effects of these conditions. Since sunflower heads, after alkaline pretreatment, cause instability to continuously operated anaerobic digesters, they should be excluded from lignocellulosic mixtures which are subjected to alkaline pretreatment, even under mild conditions. On the contrary, if not pretreated, they seem to support stability in the digesters (with a methane yield of 0.15 ± 0.015 L CH_4_ g^−1^ VS) and could be used as feedstock.

## Figures and Tables

**Table 1:** Composition of the sunflower heads and stalks (raw) as well as their solid and liquid fractions (SF and LF respectively) after alkaline pretreatment.

	Raw	SF Pretreated	LF Pretreated	Raw	SF Pretreated	LF Pretreated
**Humidity (%)**	9.46 ± 0.4	1.3 ± 1.8	-	8.1 ± 0.4	2.5 ± 1.0	-
**Total solids; ΤS (% WM)**	90.5 ± 0.4	98.7 ± 17.6	-	91.9 ± 0.3	97.5 ± 1.0	-
**Volatile solids; vs. (% WM)**	72.4 ± 0.8	87.8 ± 10.4	-	80.6 ± 0.2	83.3 ± 11.5	-
**VS (% ΤS)**	79.9 ± 0.5	88.9 ± 0.5	-	87.7 ± 0.1	85.5 ± 0.3	-
**Chemical oxygen demand; COD (SF g kg^−1^TS or LF g L^−1^)**	890 ± 26	967 ± 28	8.3 ± 0.3	1075 ± 34	1067 ± 56	6.2 ± 0.1
**ΤΚΝ (g kg^−1^TS)**	13.3 ± 0.3	13.8 ± 0.3	-	6.3 ± 0.0	5.6 ± 0.2	-
**Lipids (g kg^−1^TS)**	2.5 ± 0.1	-	-	0.99 ± 0.06	-	-
**Phenols (mg Gallic Acid L^−1^)**	-	-	214 ± 4	-	-	112 ± 2

WM: wet matter, DM: dry matter.

**Table 2:** Lignin content of raw and pretreated sunflower residues.

	Klason Lignin
	(% Raw VS)
**Heads raw matter**	10.48 ± 0.28
**Heads pretreated**	7.19 ± 0.19
**Stalks raw matter**	20.36 ± 0.66
**Stalks pretreated**	18.74 ± 0.24

**Table 3:** Elemental analysis of raw stalks and heads.

Component	Concentration (ppm)		Component	Concentration (ppm)	
	Stalks	Heads		Stalks	Heads
**Al**	1570	600	**Ga**	1	0
**Ca**	53,770	27,790	**La**	10	1
**Fe**	990	490	**Mo**	0	0
**Mg**	14,170	10,540	**Nd**	1	1
**P**	2210	2080	**Ni**	12	4
**K**	30,760	98,780	**Rb**	35	185
**Si**	19,120	2870	**Sc**	0	0
**Na**	2770	130	**Sr**	119	95
**S**	6670	5780	**Th**	0	0
**Ti**	100	50	**U**	0	0
**As**	0	0	**Sb**	0	0
**Cd**	0	0	**Hg**	0	0
**Cr**	18	0	**Br**	165	253
**Cu**	31	29	**Cs**	3	4
**Pb**	109	162	**Bi**	0	0
**Mn**	234	22	**Sm**	10	11
**V**	0	0	**W**	0	1
**Zn**	111	21	**Zr**	0	0
**Ba**	128	58	**Cl**	18,944	21,637
**Ce**	5	3	**Y**	0	0
**Co**	5	5	**Nb**	0	0

**Table 4:** Biochemical methane potential (BMP) of raw and pretreated sunflower residues. SF: solid fraction, LF: liquid fraction.

Sunflower Residues	BMP (mL CH4 g^−1^ Raw VS) Mean ± SD (± CI)			
	Raw	Pretreated Residues (Sum of SF and LF)	Pretreated Residues (SF)	Pretreated Residues (LF)
**Heads**	210.56 ± 1.97 (±2.23)	268.47 ± 3.38 (±3.83)	182.01 ± 3.17 (±3.59)	86.46 ± 6.44 (±7.29)
**Stalks**	127.98 ± 5.19 (±5.88)	168.17 ± 6.87 (±7.77)	140.11 ± 4.62 (±5.23)	28.06 ± 5.47 (±6.19)

**Table 5:** Operating conditions of Continuous Stirred Tank Reactors (CSTRs) for both phases (values are given as the average ± standard deviations; numbers in parenthesis express the 95% confidence intervals on the mean values).

	CSTR1	CSTR2	CSTR3
	**Operating Phase I: Liquid Fraction Only**		
**NaOH (g 100 g^−1^ TS)**	4	4	0
**Hydraulic Retention Time; HRT (d)**	21 ± 7	15 ± 4	16 ± 6
**Organic Loading Rate; OLR (mg L^−1^ d^−1^)**	657 ± 243 (±82)	791 ± 224 (±75)	624 ± 316 (±108)
	**Operating Phase II: Whole Slurry**		
**NaOH (g 100 g^−1^ TS)**	4	8	0
**HRT (d)**	25 ± 4	25 ± 1	25 ± 1
**OLR (mg L^−1^ d^−1^)**	2079 ± 159 (±47)	1900 ± 81 (±25)	1970 ± 0 (±0)

**Table 6:** Biogas production rate (BPR) and operational characteristics during the continuous mode of the three CSTRs (Mean ± SD (± CI)).

**Phase I**	**VSS (g L^−1^)**	**pH**	**BPR (mL L^−1^ d^−1^)**
**CSTR 1**	3.2 ± 0.4 (± 0.5)	7.38 ± 0.04	161 ± 26 (± 19)
**CSTR 2**	3.7 ± 0.4 (± 0.4)	7.42 ± 0.06	205 ± 23 (± 17)
**CSTR 3**	2.8 ± 0.3 (± 0.3)	7.30 ± 0.07	179 ± 12 (± 11)
**Phase II**	**VSS (g L^−1^)**	**pH**	**BPR (mL L^−1^ d^−1^)**
**CSTR 1**	15.1 ± 1.3 (± 1.8)	8.49 ± 0.18	unstable
**CSTR 2**	16.1 ± 1.6 (± 1.8)	7.22 ± 0.16	unstable
**CSTR 3**	16.0 ± 2.0 (± 2.8)	7.21 ± 0.05	505± 52 (± 42)
